# Circulating Brain Injury Exosomal Proteins following Moderate-to-Severe Traumatic Brain Injury: Temporal Profile, Outcome Prediction and Therapy Implications

**DOI:** 10.3390/cells9040977

**Published:** 2020-04-15

**Authors:** Stefania Mondello, Vivian A. Guedes, Chen Lai, Endre Czeiter, Krisztina Amrein, Firas Kobeissy, Yehia Mechref, Andreas Jeromin, Sara Mithani, Carina Martin, Chelsea L. Wagner, András Czigler, Luca Tóth, Bálint Fazekas, Andras Buki, Jessica Gill

**Affiliations:** 1 Department of Biomedical and Dental Sciences and Morphofunctional Imaging, University of Messina, 98125 Messina, Italy; 2 Oasi Research Institute-IRCCS, 94018 Troina, Italy; 3 National Institutes of Health, National Institute of Nursing Research, Bethesda, MD 20892, USAgillj@mail.nih.gov (J.G.); 4 Department of Neurosurgery, University of Pecs, H-7623 Pecs, Hungary; 5 János Szentágothai Research Centre; University of Pécs, H-7624 Pécs, Hungary; 6 MTA-PTE Clinical Neuroscience MR Research Group, H-7623 Pécs, Hungary; 7 Department of Psychiatry and Neuroscience, McKnight Brain Institute, University of Florida, Gainesville, FL 32606, USA; 8 Department of Biochemistry and Molecular Genetics, American University of Beirut, Beirut, Lebanon; 9 Department of Chemistry and Biochemistry, Texas Tech University, Lubbock, TX 79409, USA; 10 Cohen Veterans Biosciences, Cambridge, MA 02142, USA

**Keywords:** traumatic brain injury, biomarkers, exosomes, exosomal protein, serum, GFAP, UCH-L1, t-tau, NFL

## Abstract

Brain injury exosomal proteins are promising blood biomarker candidates in traumatic brain injury (TBI). A better understanding of their role in the diagnosis, characterization, and management of TBI is essential for upcoming clinical implementation. In the current investigation, we aimed to explore longitudinal trajectories of brain injury exosomal proteins in blood of patients with moderate-to-severe TBI, and to evaluate the relation with the free-circulating counterpart and patient imaging and clinical parameters. Exosomal levels of glial (glial fibrillary acidic protein (GFAP)) and neuronal/axonal (ubiquitin carboxy-terminal hydrolase L1 (UCH-L1), neurofilament light chain (NFL), and total-tau (t-tau)) proteins were measured in serum of 21 patients for up 5 days after injury using single molecule array (Simoa) technology. Group-based trajectory analysis was used to generate distinct temporal exosomal biomarker profiles. We found altered profiles of serum brain injury exosomal proteins following injury. The dynamics and levels of exosomal and related free-circulating markers, although correlated, showed differences. Patients with diffuse injury displayed higher acute exosomal NFL and GFAP concentrations in serum than those with focal lesions. Exosomal UCH-L1 profile characterized by acutely elevated values and a secondary steep rise was associated with early mortality (*n* = 2) with a sensitivity and specificity of 100%. Serum brain injury exosomal proteins yielded important diagnostic and prognostic information and represent a novel means to unveil underlying pathophysiology in patients with moderate-to-severe TBI. Our findings support their utility as potential tools to improve patient phenotyping in clinical practice and therapeutic trials.

## 1. Introduction

Reducing mortality and significant lifelong health loss following moderate to severe traumatic brain injury (TBI) remains a major unmet clinical goal [1,2]. In observational studies, severe TBI-related death rate is high (approximately 30%–40%), with no clear change since 1990 [3]. Among TBI survivors, nearly half of those with moderate or severe TBI require years of intensive therapy and face substantial disability and reduced life expectancy, with ensuing disruptive effects and enormous costs to the individuals as well as to their families and society [4,5]. Such dramatic/staggering burden stands in striking contrast to the lack of effective interventions. The challenge consists in the substantial injury-specific and patient-specific variability. Defining biological markers closely associated with disease pathophysiology and injury phenotype, and establishing trajectories of change during the acute phase capable of informing tailored management and acting as surrogate endpoints of treatment effect are, therefore, fundamental steps to develop therapeutics strategies to improve clinical outcomes.

Exosomes, small secreted extracellular vesicles, are emerging as a new promising diagnostic and predictive class of blood biomarkers [6,7]. They carry an array of lipids, proteins, DNA, and RNA that reflect phenotype as well as pathobiological processes of their cell of origin, and are, thereby, deeply rooted in the mechanisms underlying the disease and recovery. Further, due to their inherent features, exosomes have been shown to easily transit the blood–brain barrier (BBB) and enter into the peripheral circulation, and be elevated in various neurological diseases [8]. Extensive work has been done to explore glial (glial fibrillary acidic protein (GFAP)) and neuronal/axonal (ubiquitin carboxy-terminal hydrolase L1 (UCH-L1), neurofilament light chain (NFL), and total-tau (t-tau)) protein levels in peripheral blood after moderate and severe TBI [9,10,11,12], whereas data are lacking regarding their circulating exosomal levels and dynamics.

In the present study, we longitudinally measured serum brain injury exosomal proteins in patients with moderate-to-severe TBI using digital array technology to evaluate their temporal profile and relationship with clinical characteristics and outcome. We also investigated whether brain injury exosomal protein changes correlate with their free-circulating counterpart.

## 2. Patients and Methods

### 2.1. Study Population and Sample Collection 

This research is part of the Novel Biomarkers for Improved Characterization, Disease Tracking and Outcome Prediction in Traumatic Brain Injury, a prospective study designed to use a granular innovative multimarker strategy to advance characterization of patients with moderate to severe TBI. Patients were eligible if they were ≥ 18 years old and had a diagnosis of nonpenetrating moderate-to-severe TBI (Glasgow Coma Score (GCS) of 12 or less upon admission). Exclusion criteria were pregnancy; GCS equal to 3 associated with bilateral fixed and dilated pupils; normal head CT; and neurological comorbidities that could affect biomarker concentrations, such as neurodegenerative disorders, history of stroke, or cerebrovascular events. All patients underwent head CT examinations upon presentation and were managed according to international guidelines [13,14]. Study clinical procedures included a detailed collection of clinical data, with variables coded in accordance with The National Institute of Neurological Disorders and Stroke (NINDS) Common Data Elements (CDE) scheme. 

In the current investigation, we focused on a cohort of 21 patients, enrolled from 6 June 2018 to 22 February 2019, in whom analysis of exosomes in serum was available. The study was approved by the Local Ethics Committee (Institutional Review Board (IRB)#: IRB00003108-U Pecs, Med Ctr IRB #1; approval protocol number: 7179-PTE 2018) and written informed consent to participate in the study was obtained from next of kin. 

Venous blood samples were serially collected daily on admission and up to 5 days after initial injury. Approximately 5 mL of blood was drawn from each subject at each sample point. Blood samples were collected by venipuncture in gel separator tubes and centrifuged (4000 rpm for 10 min) at room temperature (RT) within 60 min, according to a standardized protocol (see the Supplementary Materials section). Serum was processed, aliquoted, and stored at −80 °C, pending analysis. To avoid the influence of common preanalytical factors, storage conditions including time and temperature were monitored, and specimens underwent a single freeze/thaw cycle. Exosome analysis was performed by the Tissue Injury Branch at the National Institute of Nursing Research (Bethesda, MD, USA) within 18 months from blood collection. All scientists involved in the analysis were masked to the patients’ characteristics. 

### 2.2. Extracellular Vesicles (EVs) Isolation and Characterization

EVs were isolated from 0.5 mL of frozen human serum. After samples were thawed, they were centrifuged at 3000× *g* for 15 min at 4 °C to remove debris. Samples then received ExoQuick solution (System Biosciences Inc., Mountainview, CA, USA) according to the manufacturer’s instructions. Then, the samples were incubated for 1 h at 4 °C and centrifuged at 1500× *g* for 30 min. The supernatant was aspirated after the centrifugation and the exosome pellet was resuspended in 250 µL of phosphate-buffered saline (PBS) of Dulbecco’s calcium- and magnesium-free salt solution (Sigma-Aldrich, St. Louis, MO, USA). Resuspended samples were stored at −80 °C. For particle characterization, samples were analyzed using a nanoparticle tracking analysis (NTA) software (Malvern Instruments, Malvern, United Kingdom) to determine the mean diameter (nm) and concentration (particles/mL) of EVs. 

### 2.3. Exosomal Protein Quantification

After thawing, each sample received mammalian protein extraction reagent (M-PER) to lyse exosomes (Thermo Scientific, Inc., Rockford, IL, USA), containing three times the suggested concentrations of protease and phosphatase inhibitors (cOmplete ULTRA Tablets, Millipore Sigma, Burlington, MA, USA). These suspensions were used to measure biomarker concentrations. Concentrations of GFAP, NFL, t-tau, and UCH-L1 in the cargo of EVs isolated from serum samples were measured using digital array technology (Quanterix Corporation, Lexington, MA, USA); this method uses single-molecule enzyme-linked immunoarrays (Simoa). Samples were analyzed in duplicate, with longitudinal samples of each participant measured in the same run or on the same plate. 

### 2.4. Measurement of Circulating Protein Concentrations

Serum NFL, tau, GFAP, and UCH-L1 were measured using the Simoa Neurology 4-plex assay kit (Quanterix, Lexington, MA, USA) on the Simoa HD-1 Analyzer instrument, as previously described [15].

### 2.5. Statistical Analysis 

Baseline characteristics were summarized using standard descriptive statistics, and an exploratory analysis was carried out to determine the distribution of the demographic and clinical variables. Continuous variables are presented as mean (SD) or median (interquartile range (IQR)), and categorical variables are summarized as absolute frequencies and percentages. To identify differences between groups in biomarker concentrations, Mann–Whitney *U* and Wilcoxon signed-rank tests were applied, as appropriate. The relation between biomarker concentrations and parameters for TBI severity and age were assessed using Spearman’s bivariate correlations. The non-parametric Friedman test followed by post hoc pairwise multiple comparisons (Dunn’s test) was performed to evaluate biomarker changes over time. Group-based trajectory analysis (TRAJ) was used to explore biomarker levels over time in blood using the Stata program and identify clusters of individuals following trends over time. The TRAJ procedure determines patterns in longitudinal biomarker data by assuming that the population is composed of a number of distinct subgroups that have their own unique biomarker profiles. The trajectories are identified on a likelihood basis using methods previously described [16,17]. Given the minimal detectable limit for each biomarker as well as the skewed distribution, a censored normal model was used. The number of distinct trajectories for each biomarker was determined by using a combination of the Bayesian information criterion (BIC), Akaike information criterion (AIC), and clinical judgment. Group assignment for each individual based on the posterior group membership probability was used to explore between-group differences in covariates not included in the model in separate analyses. To determine the sensitivity and specificity, a contingency table was constructed. All tests were two-sided, and significance was determined at *p* < 0.05. All statistical analyses were performed using Stata Data Analysis and Statistical Software (v.13, College Station, TX, USA).

## 3. Results

### 3.1. Description of Population

Demographic and clinical characteristics of the 21 patients included in the study are shown in Table 1. The majority of patients were males (85%) and the mean age in the study population was 52 years (range 20–77 years). The causes of TBI were mostly related to fall (62%). Out of 21 patients, 11 (52%) had a severe TBI (GCS ≤ 8) and the remaining patients a moderate TBI (GCS 9–12). Although patients with extracranial injuries were eligible for study inclusion, 67% of the population had an isolated TBI, and the median Injury Severity Score (ISS) was 16. The majority of TBI patients (52%) had mixed lesions, and 6 (29%) had cerebral edema. In all, 52% of the population were discharged to a long-term acute care facility, and 36% were discharged to an acute rehabilitation facility or home (Table 1).

### 3.2. Concentrations and Longitudinal Profiles of Serum Exosomal Brain Damage Biomarkers

**Figure 1:**
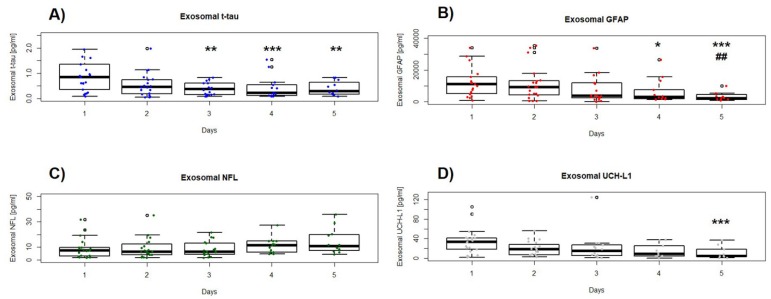
Box and whisker plots of the median concentrations of serum exosomal (**A**) total-tau (t-tau), (**B**) glial fibrillary acidic protein (GFAP), (**C**) neurofilament light chain (NFL), and (**D**) ubiquitin carboxy-terminal hydrolase L1 (UCH-L1) over time study. The upper and lower bounds of each box indicate the 75th and 25th percentiles, respectively. Within each box, the horizontal line indicates the median. Whiskers extend to 1.5 × IQR. Significant differences are indicated with * *p* < 0.05, ** *p* < 0.01, *** *p* < 0.001 vs. day 1; ^##^ *p* < 0.001 vs. day 2. For masking purposes, the displayed *y*-axis range for exosomal t-tau is 0–2.5 pg/mL (two outliers on day 1, and one on day 3 (not shown)), for exosomal GFAP is 0–40,000 pg/mL (one outlier on day 1, 3, and 5 (not shown)), and for exosomal NFL is 0–50 pg/mL (one outlier on day 1 (not shown)).

At the group level of analysis, concentrations of serum exosomal t-tau, GFAP, and UCH-L1 declined significantly (*p* < 0.001, Friedman test) over the study period (Figure 1), though the specific biomarker and individual pattern was variable (Figure S1). Both exosomal t-tau and UCH-L1 levels were substantially increased immediately after injury and quickly dropped. Median t-tau in the first 24 h was between two- and fourfold higher than at 72 to 120 h (*p* = 0.0003; Figure 1A), whereas median UCH-L1 was ≈ 8-fold more increased than at 120 h (*p* = 0.0007; Figure 1D). On the other hand, whereas median GFAP in the first 24 h was between ≈ 4- and 5-fold higher than at 96 to 120 h, concentrations remained significantly elevated (≈ 4-fold) at 48 h compared to those at 120 h (*p* < 0.0001; Figure 1B). Unlike the other markers, serum exosomal NFL showed a rising trend after injury (*p* = 0.06; Figure 1D, Table 2). 

**Figure 2:**
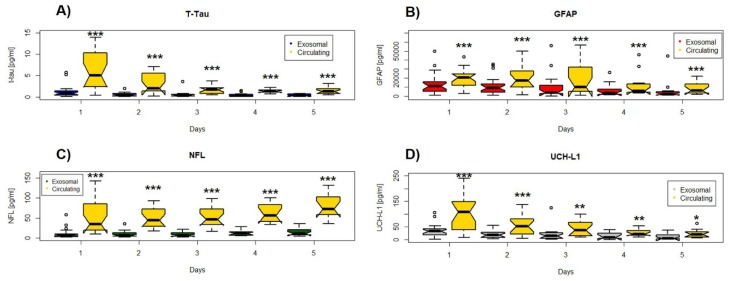
Box and whisker plots of the median concentrations of serum and exosomal and circulating (**A**) t-tau, (**B**) GFAP, (**C**) NFL, and (**D**) UCH-L1 over time of the study. The upper and lower bounds of each box indicate the 75th and 25th percentiles, respectively. Within each box, the horizontal line indicates the median. Whiskers extend to 1.5 × IQR. Significant differences are indicated with * *p* < 0.05, ** *p* < 0.01, *** *p* < 0.001.

**Figure 3:**
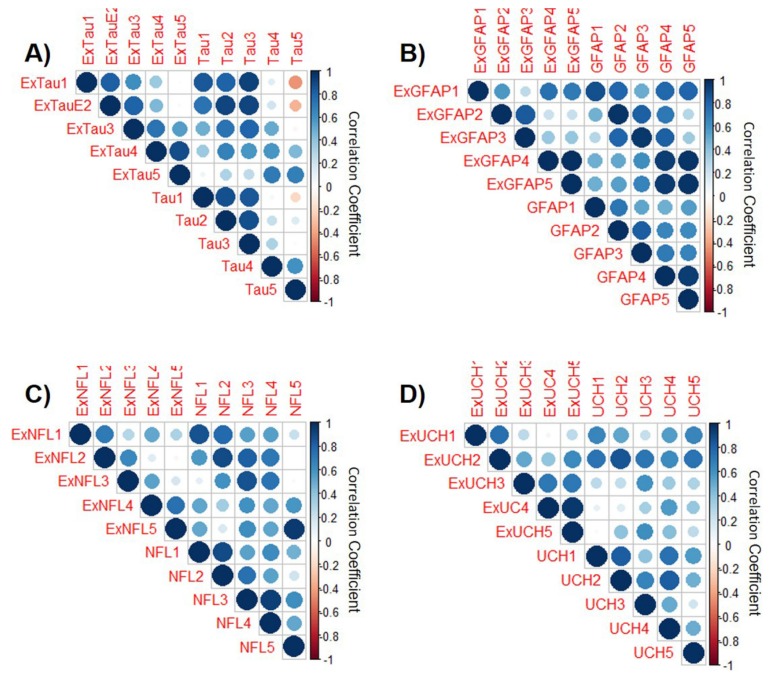
Correlation plots displaying associations between serum and exosomal (**A**) t-tau, (**B**) GFAP, (**C**) NFL, and (**D**) UCH-L1 concentration over the study. Positive correlations are displayed in blue and negative correlations in red. Color intensity and the size of the circle are indicative of the strength of correlation. In the right side of the correlogram, the legend color shows the correlation coefficients and the corresponding colors.

To address potential mechanisms underlying these dynamics, we compared exosomal brain injury protein concentrations with the related circulating levels (Figure 2). Serum protein levels were consistently higher than their exosomal counterpart and positively correlated (Figure 3). However, although the relationship remained stable and even improved over time for NFL (day 1 *r* = 0.87 and day 5 *r* = 0.95) and GFAP (day 1 *r* = 0.87 and day 5 *r* = 0.98), it appeared to decline for t-tau (day 1 *r* = 0.84 and day 5 *r* = 0.69) and UCH-L1 (day1 *r* = 0.65 and day5 *r* = 0.25) (Figure 3).

Patients with diffuse injury had significantly higher acute exosomal NFL and GFAP levels than patients with focal injury (9.88 pg/mL vs 5.49, *p* = 0.02, and 12920 pg/mL vs 6163, *p* = 0.04, respectively), but no other associations were found. Exosomal biomarker concentrations did not correlate with age or GCS, and were not associated with the mechanism of injury, ISS score, brain edema, or the need for decompressive craniectomy. Overall exosomal brain injury concentrations were not significantly different in patients requiring neurosurgical intervention compared with those not requiring such interventions.

### 3.3. Trajectory Profiles of Serum Exosomal Brain Damage Biomarkers in Moderate-to-Severe TBI Patients

**Figure 4:**
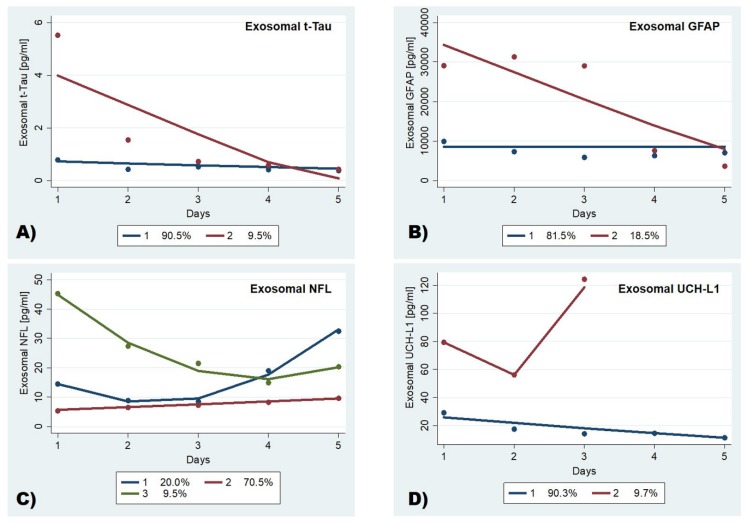
Trajectories of exosomal (**A**) t-tau, (**B**) GFAP, (**C**) NFL, and (**D**) UCH-L1. The group-based trajectory analysis procedure identified three groups for exosomal NFL, and two groups for exosomal t-tau, GFAP, and UCH-L1. Percent membership for each trajectory group is indicated.

Three distinct temporal profiles for exosomal NFL, and two distinct temporal profiles for exosomal t-tau, GFAP, and UCH-L1 were identified as the best model by trajectory analysis (TRAJ) (Figure 4). In the three-group model for NFL, the groups identified were the “low risers”, the “maximal decliners”, and the “delayed risers”. The “low risers” group, which comprised the majority of subjects (≈70.5%), had persistently low levels that slightly increased over time. The “delayed risers” started with elevated concentrations that markedly raised 3 days after injury. The “maximal decliners” group, on the other hand, started with high concentrations that dropped within 3 days, albeit remaining elevated compared with the “low risers” group (Figure 4C). 

In the two-group models for exosomal t-tau and GFAP, the groups were the “low” with consistently low levels, and the “high” with initially high levels that decreased over time (Figure 4A,B).

In the two-group model for exosomal UCH-L1, concentrations were increased early after injury in both groups. However, although one group (“minimal decliners”), which includes the majority of subjects (≈90%), started with low concentrations of UCH-L1 that declined during the first 2 days; the other group (“extreme risers”) started with substantially higher concentrations followed by a quick fall and a secondary peak, reaching exceptionally high concentrations. In this group, the trajectory ended prematurely (Figure 4D). Membership in the “extreme risers” group predicted early mortality (within 3 days), with a sensitivity and specificity of 100% (Table 3).

## 4. Discussion

In this longitudinal study of patients with moderate-to-severe TBI, we found an altered temporal profile of serum brain injury exosomal proteins following injury. Of particular interest, the dynamics and levels of exosomal and free-circulating markers, although correlated, showed differences, suggesting that they may be differently involved in disease pathogenesis and mechanisms underpinning injury and repair after TBI. In this regard, although circulating markers concentrations are thought to reflect primarily release of the proteins from dying and damaged neurons and astrocytes, multiple studies have reported that exosome biogenesis and secretion is influenced by a variety of microenvironment conditions, including oxidative stress, elevated levels of proinflammatory cytokines, and hypoxia [18,19], all hallmarks of the acute brain tissue post-severe TBI. In addition, mounting evidence indicates that exosome secretion, as well as their cargoes, are linked to changes in cell phenotypes and act as local and systemic crosstalk modulators [19]. Thus, it is plausible that exosome secreted following acute TBI could function as a means to dispose and export excess of cellular material/components—products of the disease process or of disease-induced damage [20,21] that may exert long-distance pleiotropic regulatory effects (e.g., on the immune system) and also promote neuroplasticity and regeneration after injury [22]. 

This view is further corroborated by the different correlations across markers that help unveil and enhance our understanding of the underlying pathophysiology following TBI. In our study population, correlations between free-circulating and exosomal concentration in serum were strong and improved over time for the two cytoskeletal proteins NFL and GFAP, in contrast to t-tau and especially UCH-L1, which showed weaker correlations that appreciably worsened at the late stages of injury. The most likely interpretation of these findings is that acute glial and neuroaxonal injury results in structural damage leading to (1) ensuing direct leakage of NFL and GFAP into the extracellular space and biofluids through mechanoporation, or altered plasmalemma permeability of injured cells [23,24,25], and (2) intracellular accumulation of aggregated proteins [26] that, in turn, are secreted through an exosome-mediated pathway to reduce toxicity and maintain intracellular proteostasis [21]. Taken together, these observations support the idea that free-circulating and exosomal concentrations of cytoskeletal proteins in serum are products of a common disease-induced damage that follows two distinct parallel pathways. Conversely, free-circulating and exosomal t-tau and UCH-L1 presumably may reflect and be differentially influenced by other underlying mechanisms and factors such as active secretion process of tau from neurons [27], abnormal UCH-L1 expression related to oxidative stress or other pathobiological conditions to maintain homeostasis [20,28], or, possibly, current analytical limitations due to post-translational modification. 

The complex and varied biogenesis underpinning the release of circulating exosomal brain injury proteins, along with their potential double-edged role in the contexts of the pathobiology of TBI [21,29], can provide a possible explanation for the observed absence of a correlation between exosomal markers, and injury severity and neurosurgical interventions. Alternatively, we cannot fully rule out an effect of variability in the time interval between blood draw and injury/procedures [30]. Future work is warranted. 

In this study, we demonstrated that increased exosomal NFL in blood is associated with diffuse injury. It is well known that blood NFL is a sensitive indicator of axonal injury and a promising candidate for clinical application [31]; nonetheless, this is, to our knowledge, the first study to show the potential value of its serum exosomal concentrations as a candidate tool to assess and monitor such damage. More data are needed to determine whether exosomal NFL changes might provide added value and offer complementary phenotypic and prognostic information.

Importantly, exosomal GFAP (but not circulating GFAP, data not shown) was substantially elevated in patients with diffuse injury compared to other lesion types, as assessed by computed tomography (CT). Although GFAP is regarded as a glial marker, recent lines of evidence suggest that blood GFAP concentrations might be an emerging diagnostic tool to identify white matter injury on magnetic resonance imaging (MRI) 7–18 days post-injury [32]. Our data, therefore, while complementing this evidence linking GFAP/glial damage and diffuse axonal injury, seem to indicate that exosomal levels could represent a more sensitive early indicator. In future studies, it will be interesting to explore whether and to what extent exosomal GFAP concentrations in serum correlate with advanced white matter measures, such as structural MRI and diffusion tensor imaging. In addition, our observations raise the question of whether glia-derived exosomes and their interactions with neurons play a role in the development and progression of diffuse axonal injury. Further experimental and clinical work might reveal whether this is the case.

The most clinically relevant finding was the excellent prognostic performance of the “extreme risers” exosomal UCH-L1 group to predict early mortality (within 3 days), with a sensitivity and specificity of 100%. Therefore, exosomal UCH-L1 could aid in the identification of patients deemed to die in the very early stages after injury, with direct implications for clinical practice. Furthermore, this information might be especially important in clinical trials for preventing enrolment of subjects for whom no benefit is expected from any type of intervention, which will help to maximize the chances of success [33]. Existing prognostic models including standard clinical risk factors, irrespective of the underlying pathophysiology, have proven limited utility to inform decision making [34,35,36], with only a few studies analyzing early death after a TBI [37,38]. The present work, consistent with previous research [36,39], provides evidence that blood-based biomarkers, rooted in the disease mechanisms, may yield independent and complementary prognostic information, and supports their integration into novel comprehensive multidimensional approaches for outcome prediction. However, although suggestive, our findings must be interpreted cautiously, owing to the limited number of patients (*n* = 2) who succumbed to early death in the cohort. Confirmatory studies are needed and will be an important avenue for future investigation. Moreover, future clinical translational and implementation of exosomal UCH-L1 cannot prescind from the adoption of a structured and rigorous framework to assess clinical utility, including ascertainment of cost-effectiveness and suitable health technologies, besides the clinical relevance.

We should acknowledge several limitations in this study. First, the relatively modest sample size that precluded multivariate analyses. This investigation was designed as an exploratory study, and thus larger multicenter studies are needed to replicate these findings and confirm the prognostic performance of exosomal UCH-L1 concentrations in serum. Second, we did not assess the relationship between exosomal biomarker measurements and outcome at hospital discharge, owing to the wide variation in the timing of assessment. It would be of great interest to evaluate the exosomal biomarkers’ predictive ability at standardized timepoints, including 6 and 12 months after injury, as well as to determine their relation with high intracranial pressure and hemodynamics/fluid management after injury. These are critical areas for future investigation. Third, in the current investigation, we did not isolate central nervous system (CNS)-derived exosomes, owing to the fact that the proteins we measured are primarily expressed in the nervous system and are considered as markers of brain damage. Fourth, we did not have a healthy control group. However, the longitudinal design of this study, while providing information about the dynamic change of biomarker levels after TBI, allows each patient to act as his or her own control and reduces variability [40]. Finally, the number of samples available for analysis inevitably decreased over the course of the study, which reflects the different outcomes and care pathways of the patients.

## 5. Conclusions

This study evaluated longitudinal changes in brain injury exosomal protein concentration in serum in response to acute moderate to severe TBI and the differential temporal patterns and correlations with the related free-circulating markers. Our findings strongly support the usefulness of longitudinal and comparative analyses to comprehensively understand the pathobiological mechanisms and phenotypes underlying TBI, and also determine the added and complementary value of exosomal markers. Additionally, we showed that serum exosomal UCH-L1 assessment might be a promising actionable tool to predict early mortality after injury, opening up perspectives for future applications in clinical routine and therapeutic trials. 

## Figures and Tables

**Table 1:** Characteristics of 21 patients with moderate to severe traumatic brain injury (TBI).

Variable		Value
**Age (years) Mean (SD)**		52 ± 17
**Range**		20–77
**F/M, *n* (%)**		3/18 (15%/85%)
**Ethnicity, *n* (%)**	White	21 (100)
**Race, *n* (%)**	Caucasian	21 (100)
**Mechanism of injury, *n* (%)**	Traffic accident	7 (33)
	Fall	13 (62)
	Other	1 (5)
**Time to sample withdrawal, h, median (range) ^a^**		10.8 (4.0–23.5)
**Injury severity, *n* (%)**	Moderate (GCS 9–12)	10 (48)
	Severe (GCS 3–8)	11 (52)
**ISS, median (range) ^a^**		16 (9–50)
**Marshall CT classification, *n* (%)**	Diffuse injury I	-
	Diffuse injury II	8 (38)
	Diffuse injury III	-
	Diffuse injury IV	-
	Evacuated mass lesion	12 (57)
	Non-evacuated mass lesion	1 (5)
**CT pathology, *n* (%)**	Diffuse axonal injury	1 (5)
	Extra-axial lesions only	7 (33)
	Axial lesions only	2 (10)
	Mixed lesions	11 (52)
**Cerebral edema, *n* (%) ^b^**	Yes	6 (29)
	No	15 (71)
**Decompressive craniectomy, *n* (%)**	Yes	8 (38)
	No	13 (62)
**Hospital disposition, *n* (%)**	Early death (3 days)	2 (10)
	Discharged to a long-term acute care facility	11 (52)
	Discharged to an acuterehabilitation facility	4 (19)
	Discharge home	4 (19)

CT = computed tomography; GCS = Glasgow Coma Scale; ICU = intensive care unit; ISS = Injury Severity Score; ^a^ On admission; ^b^ as assessed by CT scan.

**Table 2:** Exosomal concentrations of t-tau, GFAP, NFL, and UCH-L1 for participants.

	*n*	Median (25–75th Percentile) Range			
		t-tau, pg/mL	GFAP, pg/mL	NFL, pg/mL	UCH-L1, pg/mL
Day 1	21	0.85 (0.29–1.48) 0.08–5.74	10,944 (5029–16,550) 632.4–49,827	7.18 (2.64–12.01) 1.65–58.74	32.87 (16.44–41.79) 1.47–105.7
Day 2	20	0.46 (0.18–0.75) 0.04–1.98	9112 (4180–13,306) 553.6–35,368	6.45 (3.98–13.2) 1.47–35.28	17.8 (6.69–31.28) 2.7–56.13
Day 3	18	0.37 (0.14–0.63) 0.08–3.64	3698 (2212–13,041) 8.407–56,252	6.45 (4.25–13.3) 1.74–21.51	14.32 (4.37–28) 0.48–124.4
Day 4	13	0.22 (0.11–0.59) 0.08–1.53	2938 (1698–10,378) 1401–26,500	11.29 (6.05–14.87) 4.71–27.38	8.40 (3.95–28.51) 0.13–37.7
Day 5	12	0.28 (0.16–0.68) 0.076–0.82	2179 (1204–4894) 793.2–44,563	10.75 (7.05–20.12) 4.13–35.95	4.11 (1.87–22.94) 0.54–36.54

**Table 3:** Sensitivity and specificity of two-group exosomal UCH-L1 model for predicting early mortality.

	Early Mortality (*n* = 2)	Survivors (*n* = 19)
**Extreme risers**	2 True Positive	0 False Positive
**Low decliners**	0 False Negative	19 True Negative
Sensitivity = 100% (2/2); specificity = 100% (19/19)		

## Supplementary Materials

The following are available online at https://www.mdpi.com/2073-4409/9/4/977/s1, Figure S1: Biomarker time course profile in individual TBI patients.
