# Supplementary material for: Circulating Brain Injury Exosomal Proteins following Moderate-to-Severe Traumatic Brain Injury: Temporal Profile, Outcome Prediction and Therapy Implications
Source: Cells. 2020 Apr 15;9(4):977. doi: 10.3390/cells9040977 (PMC7227241; doi:10.3390/cells9040977)
Supplement: Supplementary file 1 [file cells-09-00977-s001.zip › Supplementary Material Final/Supplementary Material - Serum Sample Processing.pdf]

COLLECTION OF SERUM SAMPLES

- Collect blood by venipuncture into gel-separator tubes for serum first and invert tubes 5 times to activate clotting (keep at room temperature).

- Keep the serum tube samples upright and at room temperature.

- Transport samples as soon as possible to the processing laboratory.

LABORATORY PROCESSING OF SERUM SAMPLES

Serum Sample Processing

- Allow sample to clot upright at RT for 30 minutes in processing lab (45 ±15 min from time of collection), then spin at 1200 RCF (*g*) at room temperature for 15 minutes if fixed angle centrifuge rotor or 10 minutes if using a horizontal (sling) rotor. Record centrifugation time and RPM.

Note: it is important to determine in advance the RPM necessary for the specific centrifuge rotor to be used to obtain 1200 RCF.

- Using an adjustable volume reference pipette, pipette aliquots of cleared serum (500μl) into 2 ml cryovials immediately after centrifugation.

- Place in -80 freezer and record time placed.

RECORDING SAMPLE PROCESSING AND TEMPORARY STORAGE

- Place sample box into a -80°C freezer.

- Record the date and time the samples were placed in the freezer, and who processed them.

- Record the Box ID that corresponds to the sample storage box.
