# Supplementary figures and images for: Circulating Brain Injury Exosomal Proteins following Moderate-to-Severe Traumatic Brain Injury: Temporal Profile, Outcome Prediction and Therapy Implications
Source: Cells. 2020 Apr 15;9(4):977. doi: 10.3390/cells9040977 (PMC7227241; doi:10.3390/cells9040977)

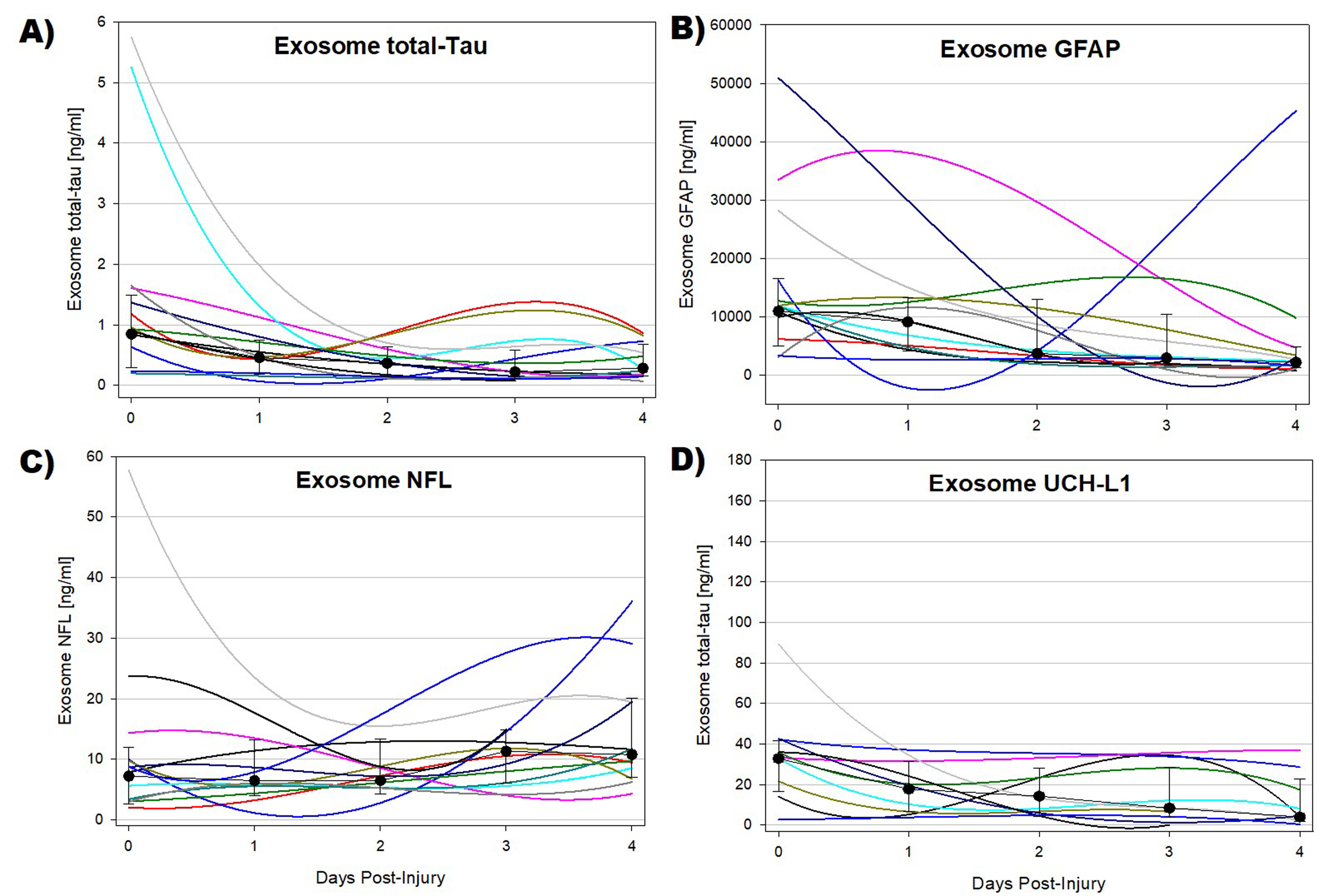

Supplement: Supplementary file 1 [file cells-09-00977-s001.zip › Supplementary Material Final/Figure 1Suppl.tif]
